# Effects of effort-reward imbalance, job satisfaction, and work engagement on self-rated health among healthcare workers

**DOI:** 10.1186/s12889-021-10233-w

**Published:** 2021-01-22

**Authors:** Jingjing Ge, Jing He, Yan Liu, Juying Zhang, Jingping Pan, Xueli Zhang, Danping Liu

**Affiliations:** 1 Department of Health Related Social and Behavioral Science, West China School of Public Health and West China Fourth Hospital, Sichuan University, Chengdu, China; 2 Gansu Provincial Maternity and Child-care Hospital, Lanzhou, China; 3 Department of Epidemiology and Biostatistics, West China School of Public Health and West China Fourth Hospital, Sichuan University, Chengdu, China; 4 Health Information Centre of Sichuan Province, Chengdu, China

**Keywords:** Healthcare workers, Self-rated health, Effort-reward imbalance, Job satisfaction, Work engagement

## Abstract

**Background:**

Healthcare workers, who protect and improve the health of individuals, are critical to the success of health systems and achieving national and global health goals. To respond effectively to the healthcare needs of populations, healthcare workers themselves must be in a good state of health. However, healthcare workers face various psychosocial pressures, including having to work night shifts, long working hours, demands of patient care, medical disputes, workplace violence, and emotional distress due to poor interactions with patients and colleagues, and poor promotion prospects. Constant exposure to these psychosocial hazards adversely impacts healthcare workers’ health. Consequently, this study aimed to examine the influence of effort-reward imbalance, job satisfaction, and work engagement on self-rated health of healthcare workers. The results would be conducive to providing policy guidance to improve the health of healthcare workers.

**Methods:**

We analysed the data of 1327 participants from The Chinese Sixth National Health and Services Survey in Sichuan Province that was conducted from August 2018 to October 2018. Structural equation modelling was used to test the hypothesized relationships among the variables.

**Results:**

Only 40.1% of healthcare workers rated their health as ‘relatively good’ or ‘good’. Effort-reward imbalance had a significant negative correlation with self-rated health (β = − 0.053, 95% CI [− 0.163, − 0.001]). The associations of effort-reward imbalance and work engagement with self-rated health were both mediated by job satisfaction (95% CI [− 0.150, − 0.050] and [0.011, 0.022]), and work engagement mediated the relationship between effort-reward imbalance and self-rated health (95% CI [− 0.064, − 0.008]).

**Conclusion:**

In order to improve the health of healthcare workers, administrators should balance effort and reward and provide opportunities for career development and training. In addition, health managers should help healthcare workers realize the significance and value of their work and keep them actively devoted to their work through incentive mechanisms.

**Supplementary Information:**

The online version contains supplementary material available at 10.1186/s12889-021-10233-w.

## Background

Healthcare workers, who protect and improve the health of individuals, are critical to the success of health systems and achieving national and global health goals [1]. Poor health among healthcare workers could affect the quality of care they provide. A previous study found that depression might affect healthcare workers’ decision making at work and relationships with colleagues and patients [2]. Likewise, poor health among healthcare workers was found to increase turnover, which not only led to a workforce shortage but also a decline in human resource quality [3, 4]. To respond effectively to the health needs of populations, healthcare workers themselves must be in a good state of health.

However, healthcare workers face various psychosocial pressures, including having to work night shifts, long working hours, demands of patient care, medical disputes, workplace violence, emotional distress due to poor interactions with patients and colleagues, and poor promotion prospects [5]. Constant exposure to these psychosocial hazards adversely impacts healthcare workers’ health. Studies have shown that healthcare workers have higher rates of suicide, burnout, minor mental disorders, hypertension, hyperlipidaemia, and asthma when compared to rates in other occupations, general workers, or the general population [6–9]. Data on healthcare workers in the United Kingdom show that sickness rates were four times higher than rates seen in other sectors [10].

Effort-reward imbalance is an important factor causing the decline in the health status of healthcare workers [11, 12]. Siegrist proposed the effort-reward imbalance model to explain this association. This model focuses on the reciprocity of extrinsic and intrinsic effort with reward [13], which consists of two core indicators: effort-reward ratio (ERR) and overcommitment (a personality characteristic). According to Siegrist et al., an imbalance between effort and reward (ERR > 1) may lead to a state of ‘active distress’ by evoking strong negative emotions [14]. This model also proposes that this process will be reinforced by overcommitment, such that overcommitted employees will respond with more strained reactions to an effort-reward imbalance compared to less committed employees. Previous studies found that ERR and overcommitment were significantly associated with adverse health outcomes among healthcare workers in Gambia, Japan, and China [11, 12, 15, 16]. Likewise, empirical studies found that ERR and overcommitment significantly predicted other negative outcomes, such as low job satisfaction and low work engagement [15, 17, 18].

Job satisfaction can be defined as a subjective feeling of how well one’s needs are being met by their job, or as ‘the extent to which people like their jobs’ [19]. Numerous studies have found that as workers’ job satisfaction decreased, their health problems increased. Job satisfaction had significant negative correlations with headaches and gastro-intestinal problems in Malaysian working women [20]. In addition, a study conducted with Chinese nurses reported that higher levels of job satisfaction were protective against developing anxiety symptoms [21]. Meanwhile, some scholars have focused on the mediating effect of job satisfaction on self-rated health; for example, Shimizu’s study identified that job stress among Japanese full-time occupational physicians contributed negatively to self-rated health indirectly through job satisfaction [22].

Work engagement is defined as a positive, fulfilling, work-related state of mind characterized by vigour, dedication, and absorption [23]. Work engagement has usually been found to be negatively associated with health problems; for example, the studies of Hakanen et al. (2012) [24] and Shu et al. (2018) [25] found that work engagement had a negative effect on depressive symptoms. Meanwhile, Shu et al.’s study found that the relationship between job stress and depressive symptoms was partly mediated by work engagement. Work engagement has been shown to impact personal outcomes such as job satisfaction [26]. For example, nurses working in Belgian hospitals who had high levels of work engagement showed high levels of job satisfaction [27]. A study among employees of a petrochemical enterprise in China suggested that interventions for improving job satisfaction may be enhanced by improving work engagement [28].

Healthcare workers’ health is critical to patients’ health and even the general population’s health. Although there have been several studies on the health of healthcare workers, they have only explored the effects of one or two variables on health, such as sociodemographic characteristics, work characteristics, effort-reward imbalance, job satisfaction, or work engagement. Few studies have combined these variables to explore how they collectively affect the health of healthcare workers, and it remains unknown how changes in the mechanisms underlying the relationships among these variables affect healthcare workers’ health. Based on the above theoretical analysis and empirical evidence, we tried to identify the associations among effort-reward imbalance, job satisfaction, work engagement, and self-rated health. The theoretical hypotheses that inform the structural equation models are shown in Table 1. The results of the current study would be conducive to providing policy guidance on improving the health of healthcare workers.

**Table 1:** Theoretical hypotheses

Hypotheses	
1. Effort-reward imbalance has a direct negative effect on self-rated health	
2. Job satisfaction has a direct positive effect on self-rated health	
3. Work engagement has a direct positive effect on self-rated health	
4. Effort-reward imbalance has a direct negative effect on job satisfaction	
5. Work engagement has a direct positive effect on job satisfaction	
6. Effort-reward imbalance has a direct negative effect on work engagement	
7. The relationship between effort-reward imbalance and self-rated health is mediated by job satisfaction	
8. The relationship between work engagement and self-rated health is mediated by job satisfaction	
9. The relationship between effort-reward imbalance and self-rated health is mediated by work engagement	

## Methods

### Study design and study population

The Chinese Sixth National Health and Services Survey (NHSS) in Sichuan Province was conducted from August 2018 to October 2018. All healthcare workers in the public medical institutions of Sichuan Province were eligible to participate in the NHSS. The study sample was selected using multistage stratified random sampling, which was the same as The Chinese Fifth National Health and Services Survey (NHSS) in Sichuan Province [29]. In the first stage, 14 cities were randomly selected from 21 cities, and a county /district was selected from each of the 14 cities. In the second stage, all third-class hospitals and some second-class hospitals were randomly selected in the 14 counties /districts. At the same time, 5 streets /towns were randomly selected from each county (district), and all community health service centres and township hospitals in each selected street and township were included in the survey medical institutions-a total of 70 community health service centers and township hospitals. In the third stage, 20 physicians and 10 nurses were randomly selected from each second-class and above hospital. At the same time, 5 physicians, 3 nurses and 2 public health professionals were randomly selected from each community health service centre and township hospital. Respondents were asked to complete the questionnaire anonymously. Informed consent was obtained from each healthcare worker following a detailed explanation about the purpose of the study. Overall, 1685 healthcare workers were investigated, of which 1327 provided valid responses (for an effective response rate of 78.80%).

### Measures

The questionnaire was developed and designed by an expert panel from the National Health Commission of the People’s Republic of China for this study.

#### Sociodemographic characteristics of healthcare workers

The sociodemographic characteristics examined included the following: gender, age (< 30, 30–39, 40–49, or ≥ 50 years), marital status (single, divorced, widowed, or married), education level (junior college or below, bachelor’s, master’s, or above), specialty (physician, nurse, or public health professional), technical title (no title, primary title, middle title, vice-senior title, or above), service years (< 5, 5–9, 10–19, 20–29, or ≥ 30 years), weekly hours at work (≤ 40, > 40), night shifts per month (none, 1–7, or ≥ 8), and grade of medical institutions (community health service centres and township hospitals, second-class hospitals, or above).

#### Effort-reward imbalance

The Effort-Reward Imbalance Scale assesses three dimensions: extrinsic effort (3 items), reward (7 items), and overcommitment (6 items). Participants responded to the items on a four-point Likert scale (1 = strongly disagree, 4 = strongly agree). To assess the degree of imbalance between high cost and low gain at work, an ERR was calculated as E/(R*C), where E was the total score of the effort dimension, R was the total score of the reward dimension, and C was the correction coefficient based on the difference in the number of numerators and denominators [30]. Here, C = 3/7 = 0.4286. An ERR value of > 1.0 indicates that the amount of effort is not rewarded adequately [19]. Higher scores represented higher overcommitment to work. Cronbach’s alpha coefficient of the scale in this study was 0.786.

#### Job satisfaction

The Job Satisfaction Scale consists of 10 items, one item for each of the following aspects of job satisfaction: opportunities to demonstrate one’s abilities, personal satisfaction, colleagues, superiors, advancement, management, training opportunities, compensation, facility, and welfare. Responses were rated on a six-point Likert scale ranging from 1 (highly disagree) to 6 (highly agree); higher scores indicated higher job satisfaction. Cronbach’s alpha coefficient of the scale in this study was 0.917.

#### Work engagement

Work engagement was measured by the Chinese version of the Utrecht Work Engagement Scale [31]. It comprises 17 items measuring three aspects of work engagement: work vigour (6 items), work dedication (5 items), and work absorption (6 items). Items were responded to using a seven-point Likert scale ranging from 0 (never) to 6 (every day) and were combined into summary scores. Higher scores indicated higher work engagement. Cronbach’s alpha coefficient of the scale in this study was 0.941.

### Outcome variable

Self-rated health status was assigned scores of 5 (good), 4 (relatively good), 3 (fair), 2 (relatively poor) and 1 (poor) by asking the participants ‘How do you feel about your health?’ Higher scores indicated better self-rated health.

### Statistical analysis

We first used descriptive statistics to examine the sociodemographic characteristics, ERR, overcommitment, job satisfaction, work engagement, and self-rated health status. Second, Pearson’s correlation coefficients were used to analyse the correlations among ERR, overcommitment, work engagement, job satisfaction, and self-rated health. Third, we used self-rated health as the dependent variable and the sociodemographic variables, ERR, overcommitment, job satisfaction, and work engagement as independent variables in a linear regression model. Fourth, a structural equation model (SEM) was employed to further test the hypothesized relationships among the study variables.

Several indicators were used to assess the fit between the current data and the hypothesized model. The goodness of fit index (GFI) > 0.9, norm fit index (NFI) > 0.9, relative fit index (RFI) > 0.9, comparative fit index (CFI) > 0.9, incremental fit index (IFI) > 0.9, and Tucker-Lewis Index (TLI) > 0.9 indicate whether the model fit was acceptable. All statistical analyses were performed using IBM SPSS version 23.0 (SPSS Inc., Chicago, IL, USA) and Analysis of Moment Structures (AMOS) version 22.0 (IBM, New York, NY, USA). Statistical significance was set at *P* < 0.05.

## Results

### Sociodemographic characteristics

The descriptive statistics of the sample are shown in Table 2. In the sample, most healthcare workers were women (63.8%) and their mean age was 37.0 years (SD = 10.0). Most were married (78.8%) and had a junior college education or below (50.3%). Physicians accounted for 58.1% of healthcare workers surveyed, followed by nurses (31.0%) and public health professionals (10.9%). Overall, 51.8% of the healthcare workers’ technical title was primary, 33.4% worked for less than 5 years. More than half of the healthcare workers worked more than 40 h per week (63.9%), 44.4% reported working night shifts 1–7 times per week, and 61.1% worked in second-class hospitals and above.

**Table 2:** Descriptive statistics (*n* = 1327)

**Characteristics**	**N**	**%**
Gender		
Male	481	36.2
Female	846	63.8
Age (years)		
< 30	361	27.2
30–39	471	35.5
40–49	335	25.2
≥50	160	12.1
Marital status		
Single, divorced, or widowed	281	21.2
Married	1046	78.8
Education level		
Junior college or below	667	50.3
Bachelor	569	42.9
Master or above	91	6.9
Specialty		
Public health professionals	144	10.9
Nurses	412	31.0
Physicians	771	58.1
Technical title		
No title	71	5.4
Primary title	687	51.8
Middle title	376	28.3
Vice-senior title or above	193	14.5
Service years		
< 5	443	33.4
5–9	388	29.2
10–19	256	19.3
20–29	171	12.9
≥30	69	5.2
Weekly hours at work		
≤40 h	479	36.1
> 40 h	848	63.9
Night shifts per month(n)		
None	394	29.7
1–7	589	44.4
≥8	344	25.9
Grade of medical institutions		
Community health service centres and township hospitals	516	38.9
Second-class hospitals and above	811	61.1
**Contents**	**Range**	**mean (SD)**
ERR	0.2–4.0	1.2 ± 0.4
Overcommitment	6–24	17.0 ± 2.7
Job satisfaction	10–60	41.6 ± 9.7
Personal satisfaction	1–6	4.2 ± 1.3
Colleagues	1–6	4.9 ± 1.0
Compensation	1–6	3.5 ± 1.5
Superiors	1–6	4.8 ± 1.2
Facility	1–6	4.1 ± 1.3
Advancement	1–6	4.0 ± 1.3
Management status	1–6	4.2 ± 1.3
Welfare	1–6	3.7 ± 1.4
Training opportunities	1–6	3.9 ± 1.3
Opportunities to demonstrate my abilities	1–6	4.3 ± 1.2
Work engagement	0–102	69.5 ± 19.8
Work vigour	0–36	23.4 ± 7.5
Work dedication	0–36	22.1 ± 6.1
Work absorption	0–36	24.0 ± 7.4
Self-rated health	1–5	3.4 ± 0.9

*ERR* Effort/reward ratio

The mean scores for ERR and overcommitment were 1.2 ± 0.4 and 17.0 ± 2.7, respectively. Most healthcare workers had an ERR higher than 1.0 (64.7%). The mean scores for job satisfaction and work engagement were 41.6 ± 9.7 and 69.5 ± 19.8, respectively. Job satisfaction regarding compensation, welfare, and training opportunities was relatively lower than the other seven aspects, with scores of 3.5 ± 1.5, 3.7 ± 1.4, and 3.9 ± 1.3, respectively. The mean score for self-rated health was 3.4 ± 0.9, and only 40.1% of healthcare workers rated their health as ‘relatively good’ or ‘good’.

### Correlations among the study variables

Pearson’s correlations among the study variables are shown in Table 3. ERR was negatively correlated with work engagement, while overcommitment was positively correlated with work engagement. ERR and overcommitment were negatively correlated with job satisfaction and self-rated health. Work engagement was positively correlated with job satisfaction and self-rated health. Job satisfaction was positively correlated with self-rated health.

**Table 3:** Correlation coefficients among the study variables

Variables	(1)	(2)	(3)	(4)	(5)
(1) ERR	**1**				
(2) Overcommitment	0.526**	1			
(3) Work engagement	−0.267**	0.171**	1		
(4) Job satisfaction	−0.537**	− 0.156**	0.525**	1	
(5) Self-rated health	−0.306**	− 0.244**	0.234**	0.314**	1

*ERR* Effort/reward ratio

**p < 0.01

### Linear regression analysis

Table 4 shows the statistically significant variables that emerged in the analysis. The results showed that two sociodemographic factors (service years and grade of medical institutions), overcommitment, job satisfaction, and work engagement were significantly associated with self-rated health. Healthcare workers with 5 to 9 years of work (β = − 0.068, *P* = 0.030) were less likely to report good self-rated health compared with those with less than 5 years of work. Healthcare workers who worked in second-class hospitals or above (β = 0.070, *P* = 0.022) were more likely to report good self-rated health than those who worked in community health service centres and township hospitals. Healthcare workers who experienced higher levels of overcommitment (β = − 0.206, *P* < 0.001) were less likely to report good self-rated health. Healthcare workers with higher levels of job satisfaction (β =0.145, *P* < 0.001) and work engagement (β =0.169, *P* < 0.001) were more likely to report good self-rated health. ERR was not significantly associated with self-rated health.

**Table 4:** Linear regression of factors significantly associated with the self-rated health

Factors	Unstandardized coefficients		Standardized coefficients	t	***P***-value	95%CI for β
	β	SE	β			
Constant	3.940	0.274	–	14.372	<0.001	(3.402,4.478)
Service years (ref:< 5)						
5–9	−0.129	0.059	−0.068	−2.172	0.030	(−0.245,-0.012)
10–19	−0.023	0.073	−0.010	− 0.310	0.757	(− 0.167,0.121)
20–29	0.055	0.092	0.021	0.593	0.553	(−0.126,0.235)
≥30	0.023	0.133	0.006	0.172	0.863	(−0.239,0.285)
Second-class hospitals and above (ref: Community health service centres and township hospitals)	0.124	0.054	0.070	2.294	0.022	(0.018,0.230)
Overcommitment	−0.067	0.011	−0.206	−6.307	< 0.001	(− 0.087,-0.046)
Job satisfaction	0.013	0.003	0.145	4.261	< 0.001	(0.007,0.019)
Work engagement	0.007	0.001	0.169	5.280	< 0.001	(0.005,0.010)

*R*^2^ = 0.168, *F* = 12.135, *p* < 0.001

### Test of study model

**Fig. 1:**
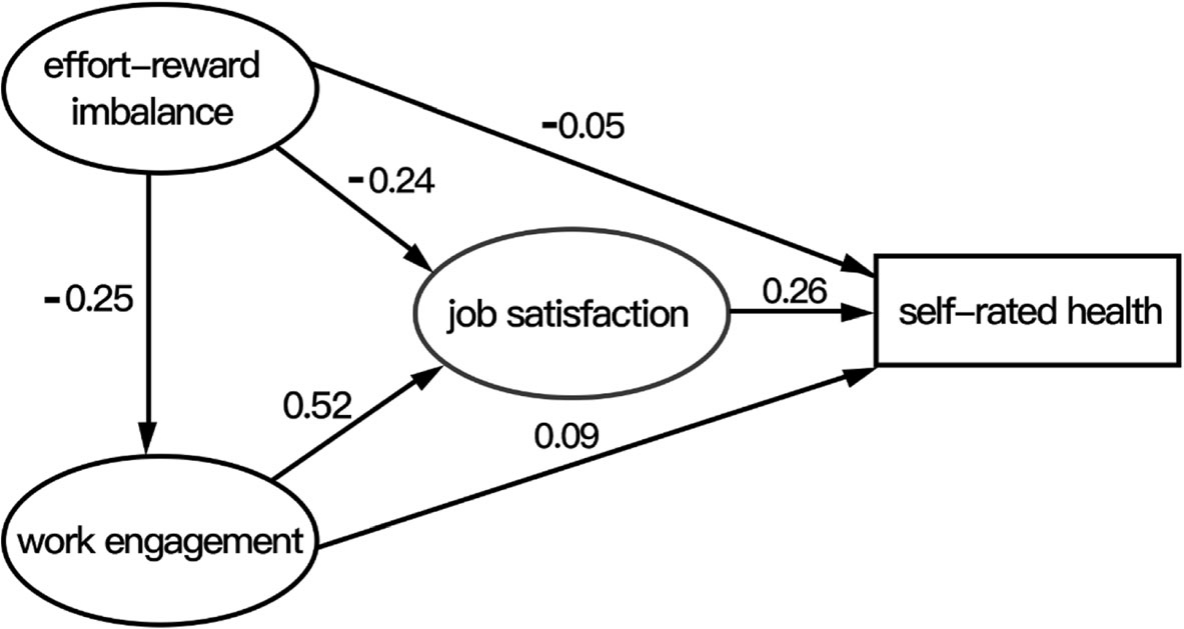
The final model and standardized model path

An SEM was used to correlate the four study variables and evaluate the relationships among them. The generalized least squares method was used to fit the data and theoretical model, and the theoretical model was corrected according to the model fit index. With the addition of the sociodemographic variables as covariates, the direction of the arrows among the core variables in the SEM remained unchanged, and the changes in the corresponding coefficients were not significant. Therefore, the sociodemographic variables were not confounding factors. The final output model is shown in Fig. 1. The overall model fit indices of the modified hypothesized model were GFI = 0.917, NFI = 0.930, RFI = 0.912, CFI = 0.937 IFI = 0.937 and TLI = 0.920. All indices met the reference value, indicating that the model fit was acceptable.

Bias-corrected bootstrap with 2000 replications using maximum likelihood estimation was employed for each path. The estimates for direct, indirect, and total effects with bias-corrected 95% CIs (confidence intervals) are shown in Table 5. Job satisfaction was significantly positively correlated with self-rated health (β = 0.255, 95% CI [0.178, 0.336]). Effort-reward imbalance was significantly negatively correlated with self-rated health (β = − 0.053, 95% CI [− 0.163, − 0.001]) and job satisfaction (β = − 0.244, 95% CI [− 0.384, − 0.092]); work engagement was significantly positively correlated with self-rated health (β = 0.086, 95% CI [0.013, 0.159]) and job satisfaction (β = 0.516, 95% CI [0.448, 0.586]); and effort-reward imbalance was significantly negatively correlated with work engagement (β = − 0.245, 95% CI [− 0.299, − 0.153]).

**Table 5:** Path coefficients between structural variables and significance test of every mediating pathway

**Model pathways**	**Estimated**	**95%CI**
**Total effects**		
Job satisfaction → Self-rated health	0.255	(0.178)–(0.336)
Effort-reward imbalance → Self-rated health	−0.169	(−0.276) -(− 0.063)
Effort-reward imbalance → Job satisfaction	−0.371	(− 0.502) -(− 0.169)
Work engagement → Self-rated health	0.217	(0.152)–(0.283)
Work engagement → Job satisfaction	0.516	(0.448)–(0.586)
Effort-reward imbalance → Work engagement	−0.245	(−0.299) -(− 0.153)
**Direct effects**		
Job satisfaction → Self-rated health	0.255	(0.178)–(0.336)
Effort-reward imbalance → Self-rated health	−0.053	(−0.163) -(− 0.001)
Effort-reward imbalance → Job satisfaction	− 0.244	(− 0.384) -(− 0.092)
Work engagement → Self-rated health	0.086	(0.013)–(0.159)
Work engagement → Job satisfaction	0.516	(0.448)–(0.586)
Effort-reward imbalance → Work engagement	−0.245	(−0.299) -(− 0.153)
**Indirect effects**		
Effort-reward imbalance → Self-rated health	−0.116	(−0.154) -(− 0.064)
Work engagement → Self-rated health	0.132	(0.087) -(0.186)
**Significance test of every mediating pathway**		
**Model pathways**		**95%CI**
Effort-reward imbalance → Job satisfaction → Self-rated health		(−0.150) -(− 0.050)
Work engagement → job satisfaction → Self-rated health		(0.011)–(0.022)
Effort-reward imbalance → Work engagement → Self-rated health		(−0.064) -(− 0.008)

*Abbreviation*: *CI* Confidence interval

Table 5 also shows the significance testing of the mediation pathways. Mediation is statistically significant if the 95% CI does not include zero. The relationships of effort-reward imbalance and work engagement with self-rated health were both mediated by job satisfaction (95% CI [− 0.150, − 0.050] and [0.011, 0.022]), and work engagement mediated the relationship between effort-reward imbalance and self-rated health (95% CI [− 0.064, − 0.008]).

## Discussion

The purpose of this study was to explore the relationships between effort-reward imbalance, job satisfaction, work engagement, and self-rated health among healthcare workers. Furthermore, the roles of job satisfaction and work engagement as mediators in the relationship between effort-reward imbalance and self-rated health were examined. This is the first study to examine the relationships among these four variables within one structural model and highlight how effort-reward imbalance, job satisfaction, and work engagement affect self-rated health among healthcare workers.

The results showed that only 40.1% of healthcare workers rated their health as ‘relatively good’ or ‘good’, which is lower than that of healthcare workers from Norway (88.1%), Germany (63.3%), Gambia (85.0%), and Brazil (65.6%) [11, 32, 33]. Additionally, this rate was lower than that of the general population in previous studies [34, 35], suggesting that self-rated health among healthcare workers in the current study was generally low. The difference in ratings may be due to medical practice being a particularly high-stress occupation in China, as healthcare workers must see many patients daily, work night shifts, have heavy workloads, and high work requirements. These factors are compounded by having to face the death and pain of patients, risk of infection, uncertainty of treatment, conflicts with patients, and lack of social support, which are huge challenges in their work [16]. It is well-documented that prolonged exposure to a stressful work environment can reduce healthcare workers’ health.

In our study, healthcare workers had lower job satisfaction regarding compensation, welfare, and training opportunities. This finding coincides with an investigation in the Chinese Province of Hubei where it was found that most healthcare workers were not satisfied with their current job and were less satisfied with the compensation packages and training opportunities [36]. The model in the current study verified that job satisfaction directly positively influenced healthcare workers’ self-rated health, as mentioned in other studies. For example, Satuf’s study suggested that high levels of satisfaction with the nature of one’s work and with one’s colleagues positively influenced physical and mental health [37].

Overall, 64.7% of healthcare workers had an ERR higher than 1.0. This result can be explained by China’s national conditions and the work characteristics of the healthcare sector. In China, healthcare workers’ existing resources are unable to meet the needs of the large patient population [38]; this situation requires healthcare workers to expend more effort to achieve organizational overall goals [39]. The results indicated that overcommitment among healthcare workers in this study was high, suggesting that they might overestimate their own abilities and put more effort into completing work that is beyond their capabilities. This study showed that effort-reward imbalance negatively influenced self-rated health; correspondingly, the same results have been obtained in studies of other occupations [12, 40]. A possible reason may be that participants believed that they receive lower rewards, such as an unsatisfactory salary and low career opportunities in relation to their efforts. If they perceive failed reciprocity between efforts and rewards, they will experience emotional distress, which in turn may cause stress-related mental and physical distress [39]. Additionally, strain reactions are reinforced by high overcommitment, increasing the risk of mental and physical illness [30].

This study also identified that the relationship between effort-reward imbalance and self-rated health was mediated by job satisfaction. The model showed that effort-reward imbalance directly negatively predicted job satisfaction, which was similar to previous studies conducted among township cadres and community health workers [17, 41]. Job satisfaction depends on the degree of disparity between the reward that employees actually receive and the reward that they expect [18]. This study suggests that lower-than-expected psychological or economic rewards for one’s efforts might cause job dissatisfaction.

In this study, scores on the dedication and absorption dimensions of work engagement were both high. High scores on these dimensions can be attributed to the specificity of the job, which requires healthcare workers to stay active, fostering dedication and high absorption [42]. The results revealed that work engagement had a direct positive effect on self-rated health. Previous research confirmed that employees with higher work engagement can recognize the value and significance of their work, devote more energy and enthusiasm to their work, and maintain good mental and physical health [43].

This study also showed that the relationship between work engagement and self-rated health was mediated by job satisfaction. Consistent with previous studies, work engagement directly positively influenced job satisfaction. Employees with high work engagement are more likely to be satisfied with their material, psychological, or self-actualization needs in the organization, and thus exhibit high job satisfaction [44]. Based on the above findings, it is evident that work engagement not only influences self-rated health directly but also exerts an influence on self-rated health indirectly through job satisfaction.

The most interesting finding of this study was that work engagement served as a mediator in the relationship between effort-reward imbalance and self-rated health. When employees experience high effort-reward imbalance, it reduces their emotional and cognitive availability, which are key to engaging in one’s work [45, 46]. Therefore, high effort-reward imbalance may lead to poor subsequent work engagement, which will cause a decline in self-rated health.

Regarding the relationship between sociodemographic characteristics and health status, participants who had been working between 5 and 9 years were less likely to report having good health compared with those who had been working less than 5 years. This finding may complement previous studies that indicated that workers employed between 5 and 9 years experienced significantly more burnout as compared to those working for less than 5 years [47], and that burnout was negatively associated with health [48]. In addition, participants who worked in second-class hospitals or above were more likely to report good self-rated health than those who worked in community health service centres and township hospitals. A possible explanation was that the tasks of healthcare workers working in community health service centres and township hospitals in China were becoming more and more onerous, they undertook a large number of basic public health services in addition to medical services, and their heavy workload led to poor self-rated health.

### Limitations of the study

There are limitations of the present study that should be considered. First, this study used a cross-sectional design, which precludes making causal conclusions. Longitudinal studies are needed to examine causal relationships among the variables. Second, we relied on self-report questionnaire data rather than conducting face-to-face investigations.

## Conclusions

This current study investigated how effort-reward imbalance, job satisfaction, and work engagement affect self-rated health among healthcare workers. The results showed that effort-reward imbalance had a significant negative correlation with self-rated health, while job satisfaction and work engagement had a significant positive correlation with self-rated health. The associations of effort-reward imbalance and work engagement with self-rated health were both mediated by job satisfaction, and work engagement mediated the relationship between effort-reward imbalance and self-rated health. The results have implications for interventions to improve the health of healthcare workers. In this regard, administrators should promote a balance between efforts and rewards. Concurrently, health managers should provide opportunities for career development and training. Moreover, health managers should help healthcare workers realize the significance and value of their work and keep them actively devoted to their work through incentive mechanisms.

## Supplementary Information

**Additional file 1.**

## Data Availability

All data generated or analysed during this study are included in this published article and its supplementary information files.

## Abbreviations

ERR: Effort-reward ratio
NHSS: National Health and Services Survey
SEM: Structural equation model
GFI: Goodness of fit index
NFI: Norm fit index
RFI: Relative fit index
CFI: Comparative fit index
IFI: Incremental fit index
TLI: Tucker-Lewis index
AMOS: Analysis of Moment Structures
SD: Standard deviation
CI: Confidence interval
